# Automatic Aortic Valve Extraction Using Deep Learning with Contrast-Enhanced Cardiac CT Images

**DOI:** 10.3390/jcdd12010003

**Published:** 2024-12-25

**Authors:** Soichiro Inomata, Takaaki Yoshimura, Minghui Tang, Shota Ichikawa, Hiroyuki Sugimori

**Affiliations:** 1 Graduate School of Health Sciences, Hokkaido University, Sapporo 060-0812, Japan; sou412flower@gmail.com; 2 Department of Health Sciences and Technology, Faculty of Health Sciences, Hokkaido University, Sapporo 060-0812, Japan; 3 Department of Medical Physics, Hokkaido University Hospital, Sapporo 060-8648, Japan; 4 Global Center for Biomedical Science and Engineering, Faculty of Medicine, Hokkaido University, Sapporo 060-8638, Japan; 5 Clinical AI Human Resources Development Program, Faculty of Medicine, Hokkaido University, Sapporo 060-8648, Japan; 6 Department of Diagnostic Imaging, Faculty of Medicine and Graduate School of Medicine, Hokkaido University, Sapporo 060-8638, Japan; 7 Department of Radiological Technology, School of Health Sciences, Faculty of Medicine, Niigata University, Niigata 951-8518, Japan; 8 Institute for Research Administration, Niigata University, Niigata 950-2181, Japan; 9 Department of Biomedical Science and Engineering, Faculty of Health Sciences, Hokkaido University, Sapporo 060-0812, Japan

**Keywords:** deep learning, convolution neural network, aortic valve

## Abstract

Purpose: This study evaluates the use of deep learning techniques to automatically extract and delineate the aortic valve annulus region from contrast-enhanced cardiac CT images. Two approaches, namely, segmentation and object detection, were compared to determine their accuracy. Materials and Methods: A dataset of 32 contrast-enhanced cardiac CT scans was analyzed. The segmentation approach utilized the DeepLabv3+ model, while the object detection approach employed YOLOv2. The dataset was augmented through rotation and scaling, and five-fold cross-validation was applied. The accuracy of both methods was evaluated using the Dice similarity coefficient (DSC), and their performance in estimating the aortic valve annulus area was compared. Results: The object detection approach achieved a mean DSC of 0.809, significantly outperforming the segmentation approach, which had a mean DSC of 0.711. Object detection also demonstrated higher precision and recall, with fewer false positives and negatives. The aortic valve annulus area estimation had a mean error of 2.55 mm. Conclusions: Object detection showed superior performance in identifying the aortic valve annulus region, suggesting its potential for clinical application in cardiac imaging. The results highlight the promise of deep learning in improving the accuracy and efficiency of preoperative planning for cardiovascular interventions.

## 1. Introduction

The World Health Organization (WHO) reported that cardiovascular disease (CVD) resulted in approximately 17.9 million deaths in 2019, accounting for 32% of all deaths that year [1]. Medical imaging has revolutionized modern medicine and healthcare, and imaging and computing technologies are becoming increasingly important for the diagnosis and treatment of CVD. Computed tomography (CT), magnetic resonance imaging (MRI), positron emission tomography (PET), single-photon emission computed tomography (SPECT), and ultrasound (US) are widely used for physiological understanding and diagnostic purposes in cardiology. In particular, CT and MRI are used to obtain specific information about the anatomy of the heart.

Contrast-enhanced cardiac CT scans are performed by injecting a contrast medium through the blood vessels. Contrast can be added to the image and specific tissues can be emphasized, making it possible to observe specific tissues and organs in greater detail. Therefore, it is used in many diseases, such as preoperative evaluation, and is used for measurement and evaluation in post-processing.

Aortic stenosis is a disease in which the aortic valve, a valve that separates the left ventricle of the heart from the aorta, becomes inoperable, making it difficult to pump blood throughout the body [2]. In Japan, it is estimated that 650,000 to 1,000,000 people aged 65 years or older suffer from aortic valve stenosis [3,4]. It has been reported that aortic stenosis has the highest incidence of occurrence (41.2%) in valvular heart disease [5]. One treatment option is aortic valve replacement (AVR). AVR is an open-heart surgery in which the heart is temporarily stopped, the heart is exposed, and the stenotic aortic valve is replaced with an artificial valve, carrying a high surgical risk for older patients, as reported in the literature, which states that 30–50% of these patients are not eligible for AVR [6].

In recent years, transcatheter aortic valve implantation (TAVI) has become a popular treatment method that is gentle on the body as it is a minimally invasive procedure that does not require sternotomy, aortic disconnection, aortotomy, or cardiopulmonary bypass [4]. Specifically, a percutaneous approach is used, a sheath is placed in the femoral artery, the valve is advanced via a guidewire to the aortic valve annulus, and the valve is deployed for treatment, allowing patients to be discharged from the hospital in an average of one to four days with unrestricted ambulation, making this treatment feasible for the elderly. Before TAVI treatment, it is necessary to measure various cardiac structures, such as the aortic root, the height from the valve ring to the coronary artery inlet, the sinus of Valsalva, and the sinotube junction (STJ), as reported in a previous study [7]. The measurement of various cardiac structures, such as the base of the aorta, the height from the valve ring to the coronary artery inlet, the sinus of Valsalva, and the sinotube junction (STJ), is necessary [8]. Computational fluid dynamics (CFD) has been extensively used to model and understand aortic dysfunctions, including aneurysms and dissections. This approach provides valuable insights into hemodynamics and mechanical stresses within the aorta, which are critical for predicting disease progression and planning interventions. These hemodynamic factors can complement structural measurements obtained from imaging, enhancing the overall accuracy and utility of preoperative planning for TAVI procedures [9,10,11]. One of these is the aortic annulus area, which is particularly crucial for these measurements as the precise measurement of the aortic annulus area is essential for several reasons, including its vital role in selecting the appropriately sized prosthetic valve for TAVI procedures, ensuring optimal valve sizing to prevent complications such as paravalvular leakage or valve migration post-implantation [12]. Additionally, it provides important information about the patient’s anatomy, influencing the choice of access route and procedural approach while contributing to the overall risk assessment for the TAVI procedure, thus helping clinicians make informed decisions about patient suitability and potential outcomes [13]. The measurement of the aortic valve annulus area is particularly crucial for TAVI planning as it provides a more comprehensive assessment of the annulus shape compared to diameter measurements alone since diameter measurements assume a circular shape, whereas area measurements account for the often elliptical nature of the annulus, leading to more accurate prosthetic valve sizing, which is essential for reducing complications such as paravalvular leakage or valve migration [14]. In this study, we focus on area measurement due to its superior ability to capture the true morphology of the annulus, although we also derive diameter estimates for comparison with traditional methods.

The measurement of the aortic valve annulus area is evaluated using CT imaging in order to place the correct size prosthetic valve. It is an important indicator because incorrect assessment may result in the selection of a different-sized valve, which can lead to serious complications. The aortic valve ring corresponds to the narrowest part of the aortic root and is the position of the plane of the virtual basal ring connecting the lowest points of the three valve leaflets [15]. However, several problems arise when calculating this. First, it is time-consuming because the measurement must be performed manually, and because it is a complex task, it is subject to inter-observer error [16], resulting in poor reproducibility.

In recent years, deep learning has been increasingly applied to medical applications, and various techniques for medical image classification [17,18], regression [19], object detection [20], and semantic segmentation [21] have been used to solve medical problems. While deep learning represents the latest approach, automatic measurement of the aortic valve annulus has been an active area of research using various methods. Traditional image processing techniques, statistical shape models, and other machine learning approaches have been explored in previous studies. These methods have included edge detection, thresholding, and semi-automatic approaches that combine algorithms with manual adjustments. Our study builds upon this existing work by applying and comparing two state-of-the-art deep learning techniques: segmentation and object detection. This approach offers potential advantages in terms of end-to-end learning, adaptability to image variations, and improved accuracy and efficiency [22,23]. By leveraging these advanced techniques, we aim to contribute to the ongoing evolution of automatic aortic valve annulus measurement methods. We focused on two deep learning techniques, segmentation and object detection. The aortic valve, also known as the aortic valve annulus region, is a valve that exists at the boundary between the left ventricle and the aorta. Therefore, the left ventricle and aorta can be obtained by segmentation, and it may be possible to automatically estimate the boundary area by extracting a point cloud. In object detection, an object detector can detect specific objects in an image or video by learning labeled images. Therefore, it may be possible to obtain the aortic valve annulus region automatically by learning labeled images of the aortic valve annulus region surrounded by a region of interest (ROI).

The purpose of this study is to evaluate and compare the performance of segmentation and object detection approaches for the automated extraction of the aortic valve annulus from contrast-enhanced cardiac CT images.

## 2. Materials and Methods

### 2.1. Subjects and Images

The data used in this study were obtained from 32 consecutive patients at Hokkaido University Hospital (approved by the Ethics Committee). These 32 cases were selected based on the following criteria: (1) patients who underwent contrast-enhanced cardiac CT, (2) CT images of sufficient quality for analysis without significant artifacts, and (3) the presence of a visible aortic valve annulus. Patients with previous aortic valve replacements or interventions were excluded, as were cases with severe image artifacts or incomplete CT datasets. These criteria were established to ensure a consistent and appropriate dataset for developing and evaluating our deep learning methods, although we acknowledge that this may limit generalizability to more complex cases. The images used were cross-sectional images of contrast-enhanced cardiac CT, reconstructed with a focused field of view on the cardiac region. The images were acquired using a 320-row multi-detector CT scanner (Aquilion ONE; Canon Medical Systems, Otawara, Japan). The scanning parameters included a reconstruction function optimized for soft tissue imaging (FC04), a tube voltage of 120 kV, and an automatic exposure control with a standard deviation of 10. The reconstructed images had a matrix size of 512 × 512 pixels, a window width (WW) of 350, and a window level (WL) of 50. This targeted reconstruction approach was chosen to optimize the visualization of cardiac structures, particularly the aortic valve annulus region.

### 2.2. Preprocessing

As a preprocessing step, we applied data augmentation techniques to increase the number of images in our dataset for analysis. This process involved generating additional training data from the original images through various transformations.

For both segmentation and object detection, we used 32 cases of contrast-enhanced cardiac CT images. Data augmentation was applied to increase the dataset size. For segmentation, images were rotated in 5° increments from −30° to 30° and scaled in 0.1 increments from 0.8 to 1.2, resulting in a 65-fold increase in the number of images.

For object detection, we created two datasets. Dataset A used the same augmentation as the segmentation dataset. Dataset B was created by applying only rotational augmentation (in 5° increments from −30° to 30°), resulting in a 13-fold increase in the number of images.

Both methods used five-fold cross-validation for analysis. The number of patients and images for each fold are summarized in Table 1, Table 2 and Table 3 for segmentation, object detection for Dataset A, and object detection for Dataset B, respectively.

### 2.3. Analysis Method

The analysis was performed in the following order: (1) segmentation, (2) estimation of aortic valve area based on segmentation results, (3) comparison of detection accuracy of object detectors, (4) aortic valve extraction using object detectors, and (5) aortic valve annulus area estimation based on object detection results. Both the segmentation and object detection methods were employed to extract and delineate the aortic valve annulus region. The segmentation approach aims to identify the boundaries between the left ventricle and aorta, from which the annulus region is derived. The object detection method directly localizes and outlines the annulus region as a region of interest within the CT images. In both cases, the goal is to obtain a precise delineation of the aortic valve annulus area, rather than specific coordinate points.

The analysis was performed in the following steps:

(i) Selection of 32 patient cases and collection of contrast-enhanced cardiac CT images.
(ii) Data augmentation techniques are applied to expand the image dataset (rotation and scaling).
(iii) Creation of supervised images for segmentation using 3D Slicer (Version 5.6.1; https://www.slicer.org/ [accessed on 30 March 2024]).
(iv) Images are segmented into five classes (left ventricle, left atrium, aorta, other cardiac structures, and background).
(v) Extraction of point clouds from the boundary between the left ventricle and aorta and determination of the optimal plane using PCA.
(vi) Projection of 3D point clouds onto a 2D plane and detection of peripheral points to identify the annulus region.
(vii) Comparison of the annulus extraction accuracy of object detectors using Dataset A (65-fold augmentation) and Dataset B (13-fold augmentation).
(viii) Automatic extraction of the aortic valve using the YOLOv2 architecture.
(ix) Evaluation of the accuracy of aortic valve annulus area estimation based on segmentation and object detection results using DSC.

#### 2.3.1. Implementation of Segmentation

**Figure 1:**
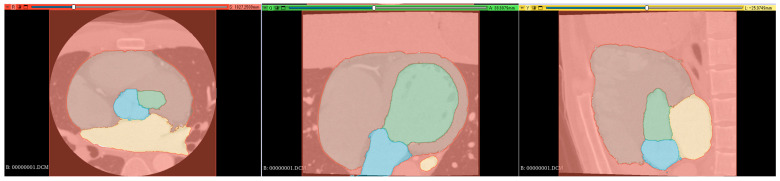
Segmentation color coding: green: left ventricle (LV), yellow: left atrium (LA), blue: aorta (Ao), brown: right atrium + right ventricle + myocardium (heart), and red: background (BG). (**Left**): axial section, (**middle**): coronal section, and (**right**): sagittal section.

Supervised images for the segmentation were created using a 3D slicer [16], which is a free open-source software for the visualization, processing, segmentation, registration, and analysis of medical, biomedical, and other 3D images and meshes. The supervised images were created through a comprehensive review process involving three radiological technologists with 25, 15, and 7 years of experience in cardiac imaging. Each technologist independently reviewed and labeled the images, manually delineating the relevant cardiac structures, including the left ventricle, left atrium, aorta, and aortic valve annulus region. Any discrepancies in labeling were discussed and resolved through consensus among the technologists. This multi-reviewer approach was chosen to ensure the accuracy and reliability of the labels, leveraging both the extensive experience of senior radiologists and the potentially fresh perspectives of more junior colleagues. Supervised images were manually labeled in axial, coronal, and sagittal orientations (Figure 1).

Segmentation resulted in five classes: left ventricle (LV), left atrium (LA), aorta (Ao), other cardiac structures (OCS) (including the right atrium, right ventricle, and myocardium), and background (BG).

#### 2.3.2. Estimation of Aortic Valve Area by Segmentation Results

**Figure 2:**
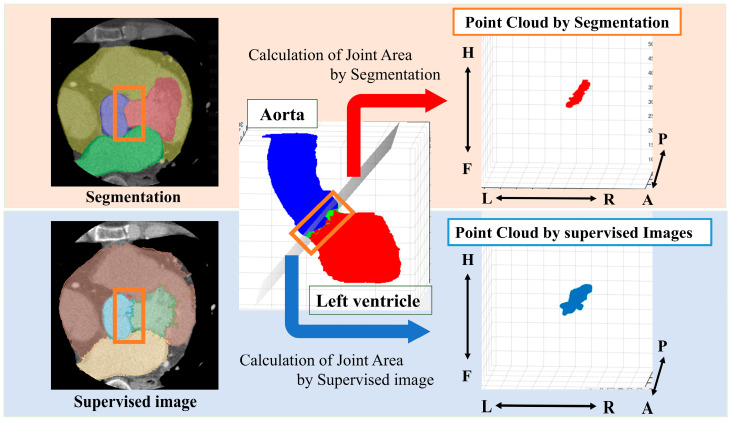
Method for obtaining a point cloud from the boundary between the left ventricle and the aorta. Orange box: segmentation boundary between aorta and left ventricle. Anatomical orientations are labeled as A (anterior), P (posterior), R (right), L (left), H (head), and F (foot).

From the segmented results, a point cloud is obtained by displaying the boundary between the LV and Ao in 3D. In this study, we define a ’point cloud’ as a set of three-dimensional coordinates representing the boundary between the left ventricle and the aorta. These points collectively form a discrete representation of the aortic valve annulus region. The point cloud is derived from either the segmentation results or the object detection outputs, depending on the method used. The point cloud is then obtained from the supervised image in the same way (Figure 2).

We performed principal component analysis (PCA) on the point cloud data to determine the best-fit plane. The normal vector of this plane corresponds to the eigenvector associated with the smallest eigenvalue from the PCA. The 3D point cloud was then projected onto this plane. To identify the aortic valve annulus contour, we applied a boundary detection algorithm to the 2D projected points, isolating the outermost points. This contour represents the boundary of the aortic valve annulus area, defined as the region enclosed by the attachment points of the aortic valve leaflets.

**Figure 3:**
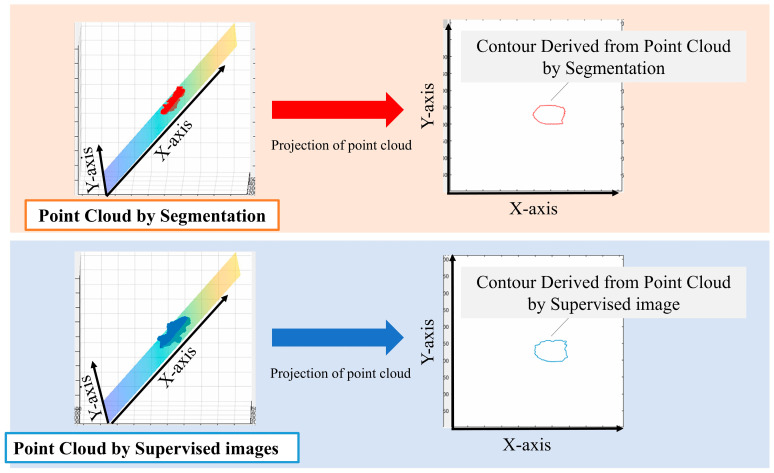
Method used for the estimation of the aortic valve ring area.

To evaluate the accuracy of our method, we compared the area enclosed by this contour with the manually segmented aortic valve annulus area from the supervised images. The segmentation and labeling process was performed by three radiological technologists with 25, 15, and 7 years of experience in cardiac imaging. Any discrepancies were resolved through a consensus discussion to minimize inter-observer variability. We calculated the DSC between these two areas to quantify their overlap. Specifically, we used PCA to find the plane that best fits the point cloud data. The normal vector of this plane corresponds to the eigenvector associated with the smallest eigenvalue from the PCA. We chose this method because it provides a mathematically optimal plane for the given points, minimizing the sum of squared distances from the points to the plane. It calculates the best plane for a point cloud from the point cloud data by finding and calculating the normal vector to that plane. All the 3D point clouds are projected onto the obtained plane and then onto a 2D plane. The periphery of the projected point cloud is assumed to be the aortic valve. Since the periphery of the point cloud calculated in Figure 3 was assumed to be the aortic valve annulus area, the accuracy was evaluated using the DSC to see how well the aortic valve annulus area calculated by segmentation matched the aortic valve area calculated from the supervised image.

#### 2.3.3. Comparison of Object Detector Detection Accuracy

First, we evaluated the valve ring extraction accuracy of the object detector using two datasets, namely, Dataset A, which was expanded 65 times by data augmentation, and Dataset B, which was expanded 13 times by rotation only. We evaluated which dataset is more accurate in extracting the valve ring of object detection.

#### 2.3.4. Aortic Valve Extraction Using Object Detectors

**Figure 4:**
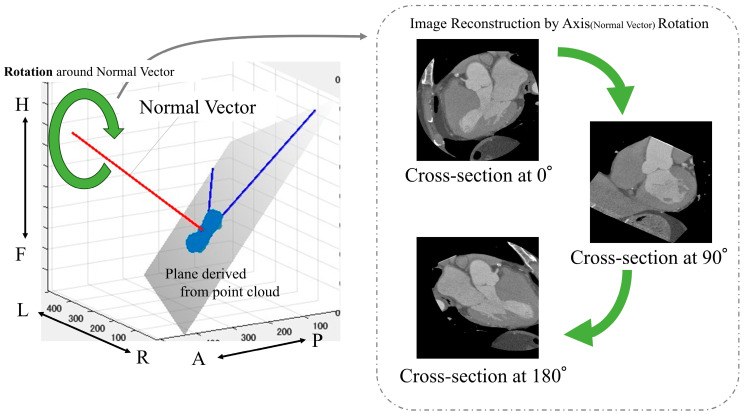
Calculation of images used in the analysis. Anatomical orientations are labeled as A (anterior), P (posterior), R (right), L (left), H (head), and F (foot).

The following is a summary of the image preprocessing used in the analysis. First, a point cloud was extracted from the boundary between the left ventricle and aorta from the segmentation results (Figure 2). Then, principal component analysis was performed on the point cloud data to calculate the optimal plane. The normal vector was obtained from the calculated plane and drawn from the center of gravity. We calculated the center of gravity of the point cloud representing the boundary between the left ventricle and aorta. Using the normal vector passing through this center of gravity as the rotation axis, we reconstructed sections rotated from 1° to 180° (Figure 4).

The aortic valve was extracted from the obtained images manually and automatically by using an object detector. With the manual method, we manually drew an ROI within the valve ring of each image that we rotated from 1° to 180° and then acquired; the image was confirmed by a clinically experienced technologist. The size of the ROI was 32 × 32 pixels for all images and was chosen based on a prior study [24] that demonstrated optimal detection performance for small structures with similar dimensions. In addition to the segmentation approach, we also employed an object detection method for aortic valve extraction. Object detection is a computer vision technique that involves both locating and classifying objects within an image. In our study, we used a deep learning-based object detector, specifically the YOLOv2 (You only look once version 2) architecture. The object detection process involves several steps. First, the CT image is input to the trained object detector. The detector then processes the image through its convolutional neural network layers. Subsequently, the network outputs bounding boxes and confidence scores for potential aortic valve locations. Finally, boxes with confidence scores above a certain threshold are considered as detected aortic valves.

**Figure 5:**
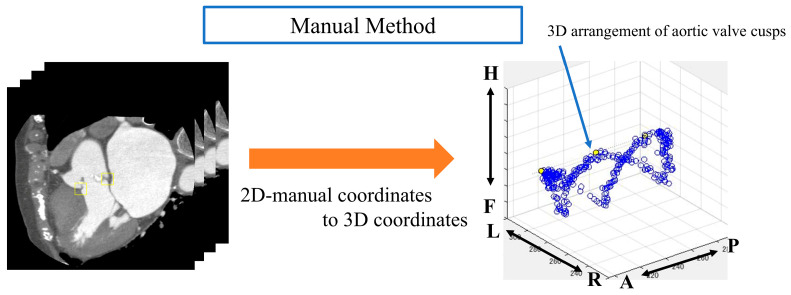
Analysis image of the manual method. Anatomical orientations are labeled as A (anterior), P (posterior), R (right), L (left), H (head), and F (foot).

**Figure 6:**
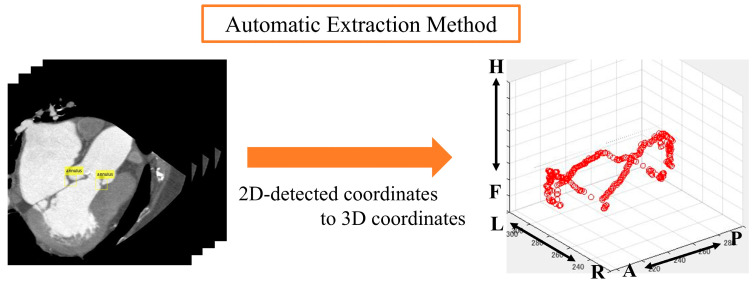
Analysis image of automatic extraction method. Anatomical orientations are labeled as A (anterior), P (posterior), R (right), L (left), H (head), and F (foot).

Automatic aortic valve extraction was performed using an object detector. After labeling all images, the center coordinates of the ROIs for both methods were displayed on a 3D plane (Figure 5 and Figure 6).

**Figure 7:**
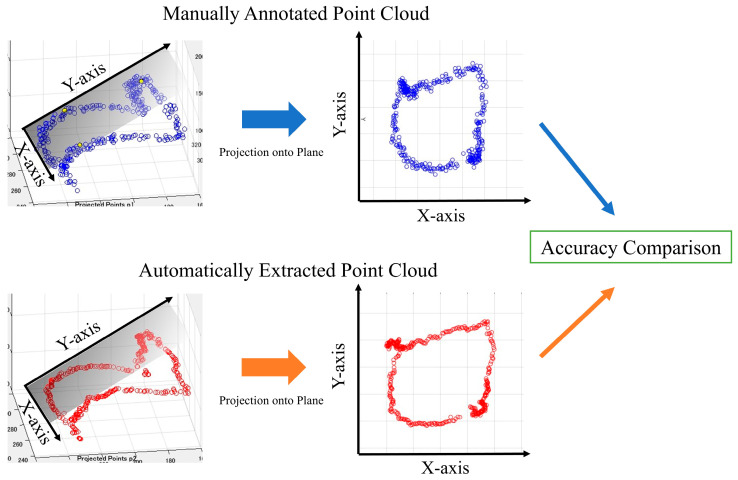
Aortic valve evaluation for both methods.

The point cloud data displayed on a three-dimensional plane was subjected to principal component analysis to obtain the optimal plane for the point cloud data. All the point clouds on the 3D plane were projected onto the obtained plane, and the periphery of the projected point clouds on the 2D plane was assumed to be the aortic valve. We then evaluated the accuracy of the aortic valve annulus area estimation for both the manual and automatic methods. This evaluation process is detailed in the following sections and illustrated in Figure 7. Since the outer circumference of the point cloud calculated from the results in Figure 7 was assumed to be the aortic valve, the accuracy was evaluated using the DSC to see how well the aortic valve annulus area calculated from the object detection results matched the aortic valve area calculated from the supervised image.

#### 2.3.5. Evaluation of Accuracy of Aortic Valve Annulus Area Estimation 

To evaluate the accuracy of the aortic valve annulus area estimation, we compared the results from both the manual and automatic methods to the ground truth data from the supervised images. The estimated aortic valve annulus area was derived from the point cloud calculated as described in the previous step. We used the DSC to quantify the agreement between the estimated area and the ground truth area. The DSC was calculated as follows:

For the purpose of this study, we defined two measures of aortic annulus diameter:

(1) Re (estimated radius): This is derived from our method’s output (either segmentation or object detection results). We calculated the area enclosed by the projected point cloud contour (as shown in Figure 7) and then determined the radius of a circle with an equivalent area. This radius is defined as Re.
(2) Rt (true radius): This is derived from the ground truth data (supervised images) using the same process as for Re.

It is important to note that Re and Rt are study-specific measures used to standardize our comparison between estimated and true aortic valve annulus areas. They are not intended to directly correspond to clinical measurements of the aortic valve annulus. To evaluate our method’s accuracy, we calculated the absolute difference |Rt − Re|. This provides a quantitative measure of how closely our estimated aortic valve annulus area matches the ground truth data.

### 2.4. Model Training Environment

#### 2.4.1. Segmentation Study and Equipment Used

**Figure 8:**
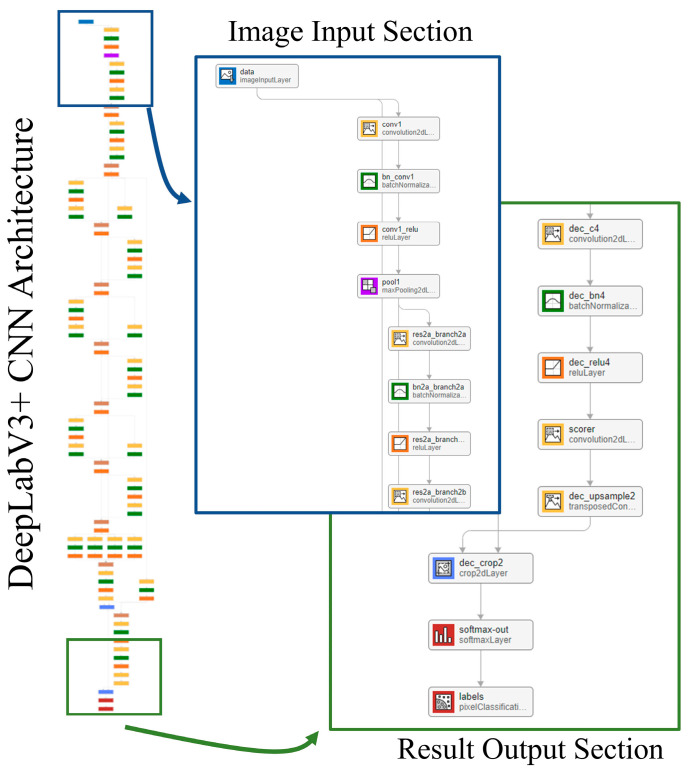
Network model.

The segmentation model takes a 2D slice from the contrast-enhanced cardiac CT image as input. The output is a pixel-wise classification of the image that is broken down into five classes: LV, LA, Ao, OCS, and BG. Each pixel in the output corresponds to one of these five classes, effectively creating a semantic map of the cardiac structures. The network model used for this segmentation is DeepLabv3+ [25], with the structure shown in Figure 8. The training conditions used for the CNN were the stochastic gradient descent with momentum (SGDM) optimizer with an initial learning rate of 0.0001, a maximum epoch of 3, a mini-batch size of 256, a learn rate drop period of 1, a learn rate drop factor of 0.3, a momentum of 0.9, and an l2 regularization of 0.005.

#### 2.4.2. Object Detection Study and Equipment Used

The object detection model also takes a 2D slice from the contrast-enhanced cardiac CT image as input. However, its output differs from the segmentation model. It produces bounding box coordinates and a confidence score for each detected instance of the aortic valve annulus region. Specifically, the output for each detection includes four values for the bounding box (x, y, width, and height) and a confidence score indicating the model’s certainty of the detection. The model used for object detection is YOLOv2, which has been pre-trained on ImageNet. The training conditions were a stochastic gradient descent with momentum (SGDM) optimizer with an initial learning rate of 0.0001, an epoch count of 3, a batch size of 256, and a momentum of 0.9. The software and equipment used are listed in Table 4.

### 2.5. Accuracy Evaluation

As a measure of accuracy, the DSC was used to estimate the agreement between the segmentation and the aortic annulus area estimated from the segmentation results and the agreement between the aortic annulus area estimated from the object detection results. The DSC expresses the ratio of the average number of elements in Datasets *A* and *B* and the number of common elements; it is obtained by the following equation:

(1) $$DSC\left(A,B\right)=\frac{2|A\;\cap B|}{\left|A\right|+|B|}$$

We evaluated the accuracy of object detectors using average precision (*AP*) as a measure of detection accuracy, which is calculated using the following equation:

(2) $$AP=\frac{TP}{TP+FP}$$

In this analysis, we used an intersection over union (IoU) threshold of 0.5 to evaluate the accuracy of our object detection method. Detections with an IoU ≥ 0.5, compared to the ground truth, were considered correct detections (true positives, *TP*), and detections with an IoU < 0.5 were considered incorrect detections (false positives, *FP*). Instances where the algorithm failed to detect an object present in the ground truth were considered missed detections (false negatives, FN). In addition, to evaluate the error rate of the object detector, we used the average miss rate (AM).

Mean and standard deviation were used as evaluation measures for segmentation and object detection.

## 3. Results

**Figure 9:**
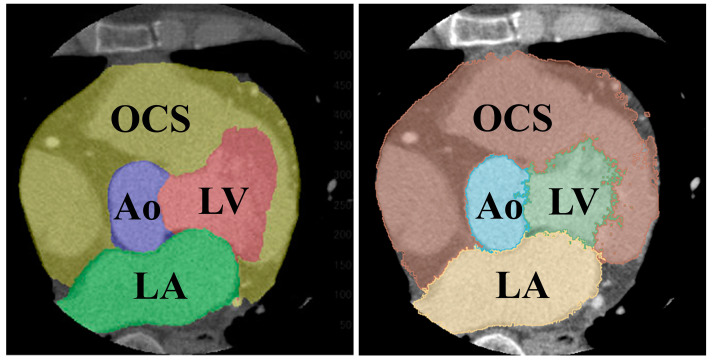
Comparison of segmented (**left**) and teacher (**right**) images of the same cross-section.

The results of the segmentation of the five classes are shown in Table 5. The average DSC was 0.907 for the LV, 0.886 for the LA, 0.880 for the heart, 0.907 for the Ao, and 0.977 for the BG. Comparing the folds of each class, fold 1 for the LV, fold 5 for the LA, fold 2 for the heart, and fold 3 for the Ao showed the highest values. A comparison of the supervised image and the segmented image of the same cross-section is shown in Figure 9. Figure 9 specifically demonstrates the segmentation results for the LV and Ao, highlighting the accuracy of our method by showing how well these segmented regions correspond to the ground truth.

The results for the aortic valve annulus area agreement using segmentation are shown in Table 6. The average DSC was 0.711, with the highest value of 0.800 for fold 3.

A comparison of the accuracy of object detection based on the two datasets is shown in Table 7 and Table 8. Table 7 shows the results for Dataset A, which was rotated and scaled 65 times using data augmentation, and Table 8 shows the results for Dataset B, which was rotated and scaled only 13 times. Dataset B has a mean AP of 0.799 and a mean AM of 0.341, with fold 5 having the highest accuracy, where AP = 0.899 and AM = 0.183.

**Figure 10:**
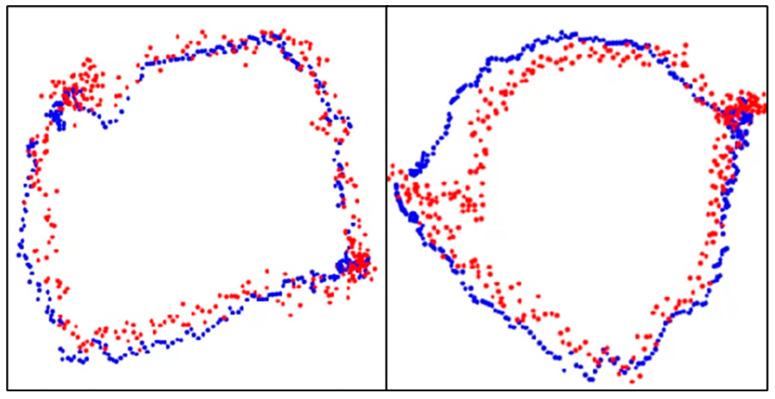
Aortic valve ring with object detection. Blue dots: aortic valve annulus from supervised images and red dots: aortic valve annulus from object detection. DSC = 0.94 (**left**) and 0.93 (**right**).

The results of aortic valve annulus area agreement using object detection are shown in Table 9. The average DSC was 0.809, with the highest value of 0.853 for model A. The dataset with the highest accuracy is shown in Figure 10.

A comparison of the estimated aortic valve ring diameters with the aortic valve ring diameters from the teacher data is shown in Table 10. In Figure 10, the blue dots represent the aortic valve annulus as determined by supervised images, while the red dots indicate the results from object detection. The DSC values of 0.94 and 0.93 demonstrate a high degree of overlap between the two methods. Table 10 compares the estimated diameters of the aortic valve annulus, showing an average error of 2.55 mm.

## 4. Discussion

From the segmented results, the LV and Ao showed an average DSC of 0.907. This indicates that the segmentation was highly accurate. On the other hand, the segmentation for the LA and heart were less accurate than the other classes, with an average DSC of 0.886 and 0.880, respectively. The lower accuracy observed for the LA and heart segments warrants further investigation. Future studies could explore whether factors such as the complex structure of the LA, including its connection to the pulmonary vein or the grouping of the right atrium, right ventricle, and myocardium into a single ‘heart’ segment, contribute to these differences in accuracy. A more detailed analysis of segmentation errors, perhaps including 3D visualization of misclassified regions, could provide insights into specific anatomical challenges [26]. Additionally, experimenting with different class definitions or more fine-grained segmentation targets might help to identify the optimal balance between segmentation detail and accuracy.

Our segmentation results for the LV and Ao demonstrate high accuracy, with a mean DSC value of 0.907 for both structures. These values are comparable to or exceed those reported in previous studies. For instance, our accuracy surpasses that reported by Finnegan et al. [27] for non-contrast chest CT images (LV: 0.88 and Ao: 0.78) and is similar to that observed by Luo et al. [28] for CT images (LV: 0.91 and Ao: 0.92). However, when compared to studies using larger datasets of contrast-enhanced cardiac CT, such as Wang et al. [29] and Sharkey et al. [30], our results show slightly lower accuracy, particularly for the Ao. This difference highlights a key limitation of our study: the relatively small sample size. The impact of sample size on segmentation accuracy is well-documented in the literature on deep learning [23]. Larger datasets typically lead to more robust and generalizable models. In our case, increasing the number of subjects could potentially improve our segmentation accuracy, particularly for more complex structures like the Ao.

Aortic valve annulus area extraction using the segmentation results showed a mean DSC of 0.711. Although the mean DSC was moderately high, there was considerable variation among the datasets, which may be due to the analysis method. Based on the segmentation results, a point cloud was extracted, and the aortic valve was defined by projecting the 3D-displayed point cloud onto what we considered the optimal plane. However, it is unclear whether the calculated optimal plane truly represents the location of the aortic valve. In addition, since the segmentation results were used as-is, any segmentation errors could propagate into the annulus extraction, leading to lower accuracy and greater variability.

We also compared the valve annulus extraction accuracy of the object detectors using two datasets. The results show that Dataset A, which underwent more extensive data augmentation (including scaling and rotation), achieved higher accuracy in terms of both mean AP and mean AM, compared to Dataset B, which only underwent rotational augmentation. The improved performance with a more comprehensive augmentation strategy suggests that increasing the diversity of training data enhances the model’s ability to handle variations in anatomy and imaging conditions. While these findings are secondary to our main objective of comparing segmentation and object detection methods, they underscore the importance of robust preprocessing in medical image analysis pipelines. Investigating other augmentation techniques, such as brightness and contrast adjustments or synthetic data generation via GANs, could further improve object detection performance.

Aortic valve annulus area extraction using object detection resulted in an average DSC of 0.809. Although aortic valve annulus regions were extracted with relatively high accuracy, some data were not extracted correctly depending on the dataset. In particular, images reconstructed from 1° to 180° often depicted two valve annuli, and while our study did not include patients with previously implanted prosthetic valves, distinguishing between multiple annuli could be challenging. Future research should investigate whether the accuracy of such cases can be improved, especially in patient populations with prosthetic valves or significant calcifications.

The average DSC values for the agreement of the aortic valve annulus area using segmentation compared to object detection were 0.711 and 0.809, respectively, indicating that object detection produced a more accurate estimation. A DSC above 0.8 generally indicates good agreement in medical image segmentation tasks [31]. This difference can be attributed to the fundamental approaches of the two methods. The segmentation-based approach relies on accurately delineating larger structures (the LV and Ao) and inferring the annulus from the boundary between them. Errors in either of these regions can propagate to the annulus estimation. In contrast, the object detection method focuses directly on the annulus region, potentially making it more robust to local image ambiguities.

The object detection method also demonstrated strong performance in absolute terms. Specifically, it achieved a mean DSC of 0.809 for aortic valve annulus area estimation, indicating a high degree of overlap with ground truth regions. Additionally, it showed a mean AP of 0.852 and a mean AM of 0.260. While these results are promising, clinical validation is needed to confirm the method’s utility in preoperative planning for procedures such as TAVI.

Since this method is unique, there are no directly comparable studies or reference indices for its accuracy. To contextualize our results, we compared them to DSC values reported in other medical imaging studies (e.g., liver tumor segmentation [32] and bladder cancer segmentation [33]). While our current DSC values are below 0.9, some data have been found to exceed this threshold, suggesting room for improvement and potential clinical relevance in the future.

We also estimated the aortic valve annulus diameter and found a mean error of 2.55 mm compared to the ground truth. Given that prosthetic valve sizes are typically defined in 3 mm increments, a mean error under 3 mm suggests the potential utility of our approach in assisting with valve selection.

A limitation of our study is that the dataset did not include patients undergoing TAVI. Expanding the dataset to include TAVI cases is an essential future step. Furthermore, there is currently no established clinical threshold for acceptable DSC values in annulus extraction, and the lack of directly comparable studies makes it difficult to determine the clinical significance of our results. Although we visually identified the annulus to create the training data, our evaluation did not involve comprehensive 3D confirmation after extraction. Future work should include more thorough visual validation, ideally involving clinical experts, and possibly integrate both 3D and 2D confirmation steps.

In summary, our study demonstrates the potential of both segmentation and object detection approaches for aortic valve annulus identification, with object detection showing particular promise in terms of accuracy. While our current results are encouraging, the path to clinical application will require addressing limitations related to dataset size, anatomical complexity, calcifications, and clinical validation thresholds. By refining these approaches and expanding the dataset, we could move closer to establishing a robust method capable of assisting clinicians in procedures like TAVI.

Future work building on these findings could further bridge the gap between computational performance and clinical utility. Future research should focus on validating the method in larger datasets and exploring its application in other cardiovascular imaging tasks. These efforts will aim to ensure the robustness of the method across diverse patient populations and extend its applicability to broader clinical contexts, potentially benefiting a wider range of cardiovascular image preprocessing.

## 5. Conclusions

This study demonstrated the effectiveness of object detection over segmentation for aortic valve annulus extraction. These findings suggest its potential utility for clinical applications, particularly in TAVI planning.

## Figures and Tables

**Table 1:** Number of patients and number of images for training and testing for each fold (segmentation).

	Training (Number of Patients)	Test (Number of Patients)	Training DA (Number of Images)	Test (Number of Images)
fold 1	25	7	915,330	3712
fold 2	25	7	901,680	3922
fold 3	26	6	947,570	3216
fold 4	26	6	930,930	3472
fold 5	26	6	930,930	3472

**Table 2:** Number of patients and number of images in training and test for each fold in Dataset A.

	Training (Number of Patients)	Test (Number of Patients)	Training DA (Number of Images)	Test (Number of Images)
fold 1	25	7	855,000	2520
fold 2	25	7	855,000	2520
fold 3	26	6	889,200	2160
fold 4	26	6	889,200	2160
fold 5	26	6	889,200	2160

**Table 3:** Number of patients and number of images in training and test for each fold in Dataset B.

	Training (Number of Patients)	Test (Number of Patients)	Training DA (Number of Images)	Test (Number of Images)
fold 1	25	7	171,000	2520
fold 2	25	7	171,000	2520
fold 3	26	6	177,840	2160
fold 4	26	6	177,840	2160
fold 5	26	6	177,840	2160

**Table 4:** Software and Equipment Used.

Environment	Content
Software	MATLAB (2022b; Mathworks, Carlsbad, CA, USA)
OS	Windows 10
CPU	Intel core i9-10980XE 3.0 GHz
GPU	NVIDIA RTX A6000 48 GB × 2
Memory	DDR4 2933 Quad-Channel 64 GB

**Table 5:** Accuracy of Segmentation.

	Fold 1	Fold 2	Fold 3	Fold 4	Fold 5	Mean
LV	0.917	0.904	0.897	0.904	0.912	0.907 ± 0.01
LA	0.877	0.877	0.896	0.878	0.903	0.886 ± 0.01
OCS	0.875	0.887	0.881	0.878	0.880	0.880 ± 0.004
Ao	0.918	0.898	0.924	0.886	0.907	0.907 ± 0.02
BG	0.975	0.977	0.978	0.980	0.977	0.977 ± 0.002

**Table 6:** Aortic valve ring area agreement for segmentation.

	Fold 1	Fold 2	Fold 3	Fold 4	Fold 5	Mean
DSC	0.729	0.646	0.800	0.726	0.664	0.711 ± 0.06

**Table 7:** Object detection accuracy for Dataset A.

	Fold 1	Fold 2	Fold 3	Fold 4	Fold 5	Mean
AP	0.881	0.857	0.828	0.782	0.914	0.852 ± 0.05
AM	0.222	0.252	0.306	0.371	0.151	0.260 ± 0.08

**Table 8:** Object detection accuracy for Dataset B.

	Fold 1	Fold 2	Fold 3	Fold 4	Fold 5	Mean
AP	0.833	0.792	0.726	0.744	0.899	0.799 ± 0.07
AM	0.301	0.351	0.454	0.414	0.183	0.341 ± 0.11

**Table 9:** Aortic valve annulus area agreement for object detection.

	Fold 1	Fold 2	Fold 3	Fold 4	Fold 5	Mean
DSC	0.853	0.726	0.848	0.777	0.847	0.809 ± 0.06

**Table 10:** Comparison of aortic valve ring diameters.

	Fold 1	Fold 2	Fold 3	Fold 4	Fold 5	Mean
|Rt − Re| [mm]	2.01	4.33	2.04	2.64	1.52	2.55 ± 1.09

## Data Availability

The models created in this study are available upon request from the corresponding author. The source code of this study is available at https://github.com/MIA-laboratory/AorticValveExtraction/ (accessed on 1 October 2024).
